# Dynamic circadian fluctuations of glycemia in patients with type 2 diabetes mellitus

**DOI:** 10.1186/s40659-022-00406-1

**Published:** 2022-12-02

**Authors:** Manuel Vásquez-Muñoz, Alexis Arce-Álvarez, Cristian Álvarez, Rodrigo Ramírez-Campillo, Fernando A. Crespo, Dayana Arias, Camila Salazar-Ardiles, Mikel Izquierdo, David C. Andrade

**Affiliations:** 1 Exercise Applied Physiology Laboratory, Centro de Investigación en Fisiología Y Medicina de Altura, Departamento Biomedico, Facultad de Ciencias de La Salud, Universidad de Antofagasta, Antofagasta, Chile; 2 Clínica Santa María, Santiago, Chile; 3 Navarrabiomed, Hospital Universitario de Navarra (UHN), Universidad Pública de Navarra (UPNA), IdiSNA, Pamplona, Navarra Spain; 4 Escuela de Kinesiología, Facultad de Salud, Universidad Católica Silva Henríquez, Santiago, Chile; 5 Exercise and Rehabilitation Sciences Laboratory, School of Physical Therapy, Faculty of RehabilitationSciences, Universidad Andres Bello, Santiago, Chile; 6 Departamento de Gestion Y Negocios, Facultad de Economía Y Negocios, Universidad Alberto Hurtado, Santiago, Chile; 7 Departamento de Biotecnología, Facultad de Ciencias del Mar Y Recursos Biológicos, Universidad de Antofagasta, Antofagasta, Chile; 8 CIBER of Frailty and Healthy Aging (CIBERFES), Instituto de Salud Carlos III, Madrid, Spain

**Keywords:** Glycemia, Diabetes mellitus, Circadian rhythm, Oscillations, Continuous glucose monitoring

## Abstract

**Background:**

Diabetes mellitus (DM) has glucose variability that is of such relevance that the appearance of vascular complications in patients with DM has been attributed to hyperglycemic and dysglycemic events. It is known that T1D patients mainly have glycemic variability with a specific oscillatory pattern with specific circadian characteristics for each patient. However, it has not yet been determined whether an oscillation pattern represents the variability of glycemic in T2D. This is why our objective is to determine the characteristics of glycemic oscillations in T2D and generate a robust predictive model.

**Results:**

Showed that glycosylated hemoglobin, glycemia, and body mass index were all higher in patients with T2D than in controls (all p < 0.05). In addition, time in hyperglycemia and euglycemia was markedly higher and lower in the T2D group (p < 0.05), without significant differences for time in hypoglycemia. Standard deviation, coefficient of variation, and total power of glycemia were significantly higher in the T2D group than Control group (all p < 0.05). The oscillatory patterns were significantly different between groups (p = 0.032): the control group was mainly distributed at 2–3 and 6 days, whereas the T2D group showed a more homogeneous distribution across 2–3-to-6 days.

**Conclusions:**

The predictive model of glycemia showed that it is possible to accurately predict hyper- and hypoglycemia events. Thus, T2D patients exhibit specific oscillatory patterns of glycemic control, which are possible to predict. These findings may help to improve the treatment of DM by considering the individual oscillatory patterns of patients.

## Introduction

Diabetes mellitus (DM) can be classified as type I DM (T1D) and type II DM (T2D) according to its etiology [1]. Public spending associated with DM in the United States reached $237 billion in 2017, and approximately 1.6 million deaths were reported in 2016 because of DM, which is now the seventh leading cause of death worldwide [2, 3]. Sustained hyperglycemia is associated with an increased risk of both micro-and macrovascular complications and can lead to retinopathy, kidney failure, stroke, and even lower limb amputation [2–5]. Measurement of glycosylated hemoglobin (HbA1c) is a standard method to monitor average blood sugar levels in patients with DM during the previous 2–3 months and has the potential to predict the risk of long-term DM complications [6–9]. However, because HbA1c is a long-term metric associated with the life-span of red blood cells, it does not accurately reflect intraday oscillations in glycemic control (dysglycemia [higher and lower levels of glucose during the day and night phases]), which are very relevant for the vascular complications of DM [10, 11].

Type 2 DM is characterized by hyperglycemia, increased HbA1c, and overweight [12, 13]. Importantly, it has been proposed that the early detection of dysglycemic episodes (acute and chronic glucose excursions) might help improve outcomes in patients with DM [14, 15]. Nevertheless, while T2D can be treated with diet, exercise, and medication, there is no accurate classification of glycemic oscillations in patients with T2D. Prospective studies have demonstrated that dysglycemias can precede a diagnosis of DM by months to years, [16, 17] and the correct identification and classification of glycemic oscillations during the day and night phases could guide early treatment, which may have important implications for preserving endogenous insulin secretion and preventing T2D complications [18].

Continuous glucose monitoring (CGM) provides real-time glucose measurements, allowing for the reliable determination of hypoglycemia/hyperglycemia episodes and, consequently, glycemic variability over several days, weeks, or even months [19, 20]. We recently characterized and classified glycemic oscillations in patients with T1D through CGM [15], finding that glycemic variability has different oscillatory patterns that are not entirely stochastic and exhibit circadian rhythmicity [21, 22]. As far as we know, there is no accurate classification of glycemic oscillations in T2D. Accordingly, the present study aimed to determine the characteristics of glycemic fluctuations in T2D and test whether they could be predicted over time in patients.

## Results

### Baseline characteristics of participants

The baseline characteristics of the study groups are described in Table 1. No significant differences were found in age, weight, or height between groups; however, BMI was significantly higher in the T2D group than in the control group. Based on the BMI, patients in the T2D group were classified as overweight (cut off > 25 kg/m^2^) [29]. The different insulin drugs used by the T2D group (rapid-acting insulin, long-acting insulin, and other hypoglycemic drugs) are also shown in Table 1.

**Table 1:** Baseline characteristics of individuals with T2D and controls

	Control n = 28 (53.84%)	T2D n = 24 (46.15%)	*P-value*
Age (years)	64.66 ± 18.31	69.96 ± 11.50	0.224
Weight (kg)	70.52 ± 7.25	71.21 ± 9.43	0.189
Height (m)	1.69 ± 0.08	1.62 ± 0.08	0.051
BMI (kg/m^2^)	**24.59 ± 2.04**	**27.08 ± 4.73**	**0.013**
Duration of diabetes (years)	–	32.25 ± 10.52	–
Rapid-acting insulin			
Novorapid	–	n = 2 (8.33%)	
Humalog	–	n = 4 (16.66%)	
Actrapid	–	n = 3 (12.50%)	
Long-acting insulin			
Lantus	–	n = 13 (54.16%)	
Tresiba	–	n = 0	
Toujeo	–	n = 0	
Levemir	–	n = 1 (8.33%)	
Insulatard	–	n = 13 (54.16%)	
Other hypoglycemic drugs			
Metformin	–	n = 15 (62.5%)	
Minidiab	–	n = 1 (4.16%)	
Janumet	–	n = 2 (8.33%%)	
Glibenclamide	–	n = 1 (4.16%)	
Januvia	–	n = 10 (41.66%)	

Data are shown as mean ± standard deviation (SD). *BMI* body mass index. Bold letters denote significant differences (p < 0.05) between groups

### Glycemic characteristics and stochastic/deterministic glycemic variability patterns

**Fig. 1:**
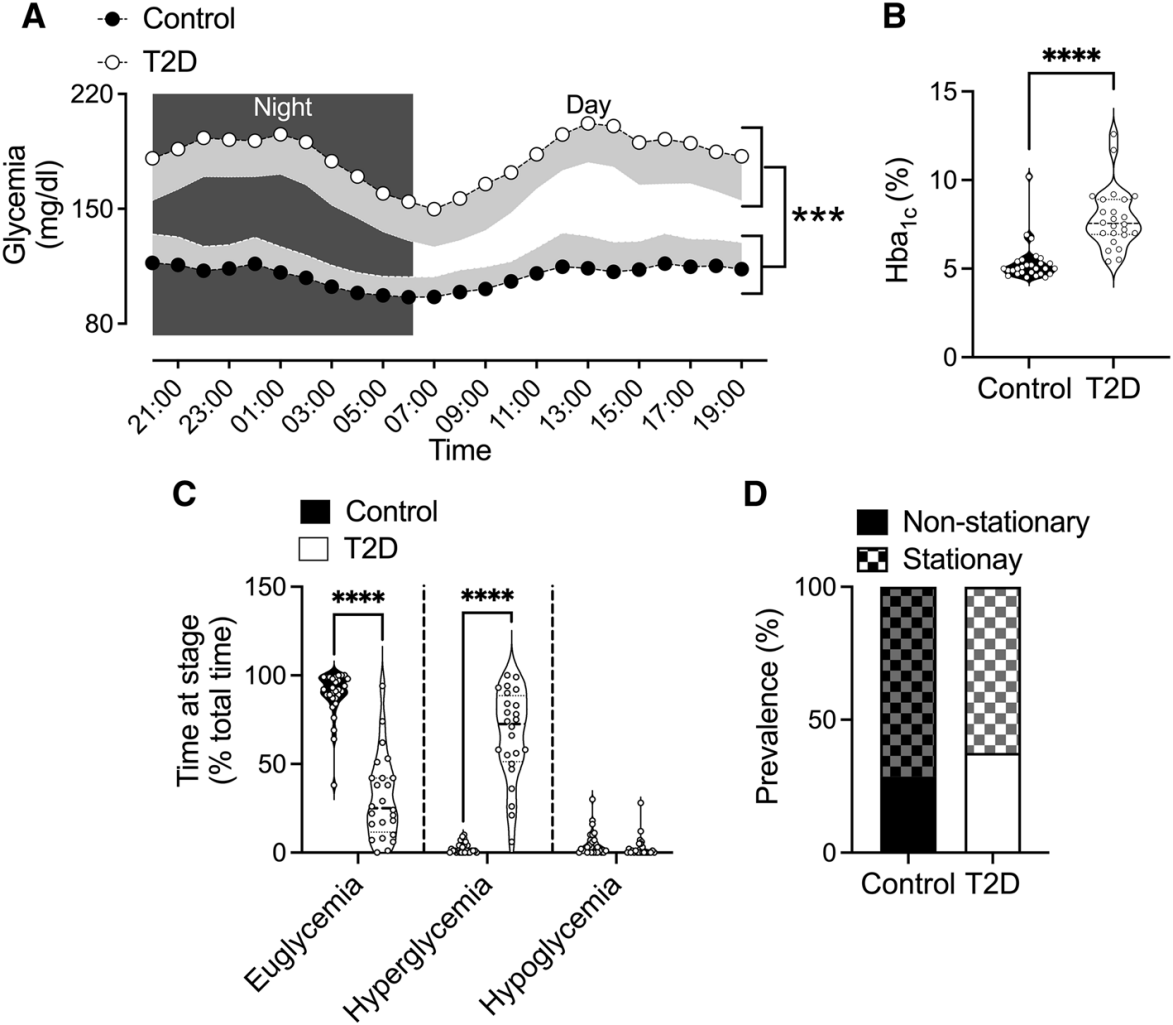
Glycemic status in patients with type 2 diabetes and control participants. **A** Circadian rhythm and oscillatory pattern of glycemia in patients with T2D and control participants. **B** HbA1c values in T2D patients and Control participants; **C** time at the stage of glycemic control (euglycemic, hyperglycemic, and hypoglycemic). The prevalenceence of stationary and non-stationary glycemic variability patterns, was determined through the Dickey-Fuller test. ***, p < 0.001; ****, p < 0.0001. Control, n = 24; T2D, n = 28

Concomitants with overweight, glycemia, and HbA1c levels were significantly greater in the T2D group than in the control group during the day and night phases (average between day and night, p < 0.001) (Fig. 1A, B).

Figure 1C shows the two study groups' time in euglycemia, hyperglycemia, and hypoglycemia. The T2D group spent significantly less time in euglycemia than the control group (p < 0.0001), whereas the opposite was observed for the time in hyperglycemia (p < 0.0001). No difference between groups was found for the time in hypoglycemia (p = 0.628).

Dickey-Fuller test revealed that 62.5 and 37.5% of T2D; and 74.1 and 25.9% of control participants displayed a stationary (deterministic) and non-stationary (stochastic) glycemia oscillatory variability, respectively (Fig. 1D). However, the differences were not significantly different (Chi-squared: 3.84; p = 0.46).

### Glycemic variability in patients with T2D

**Fig. 2:**
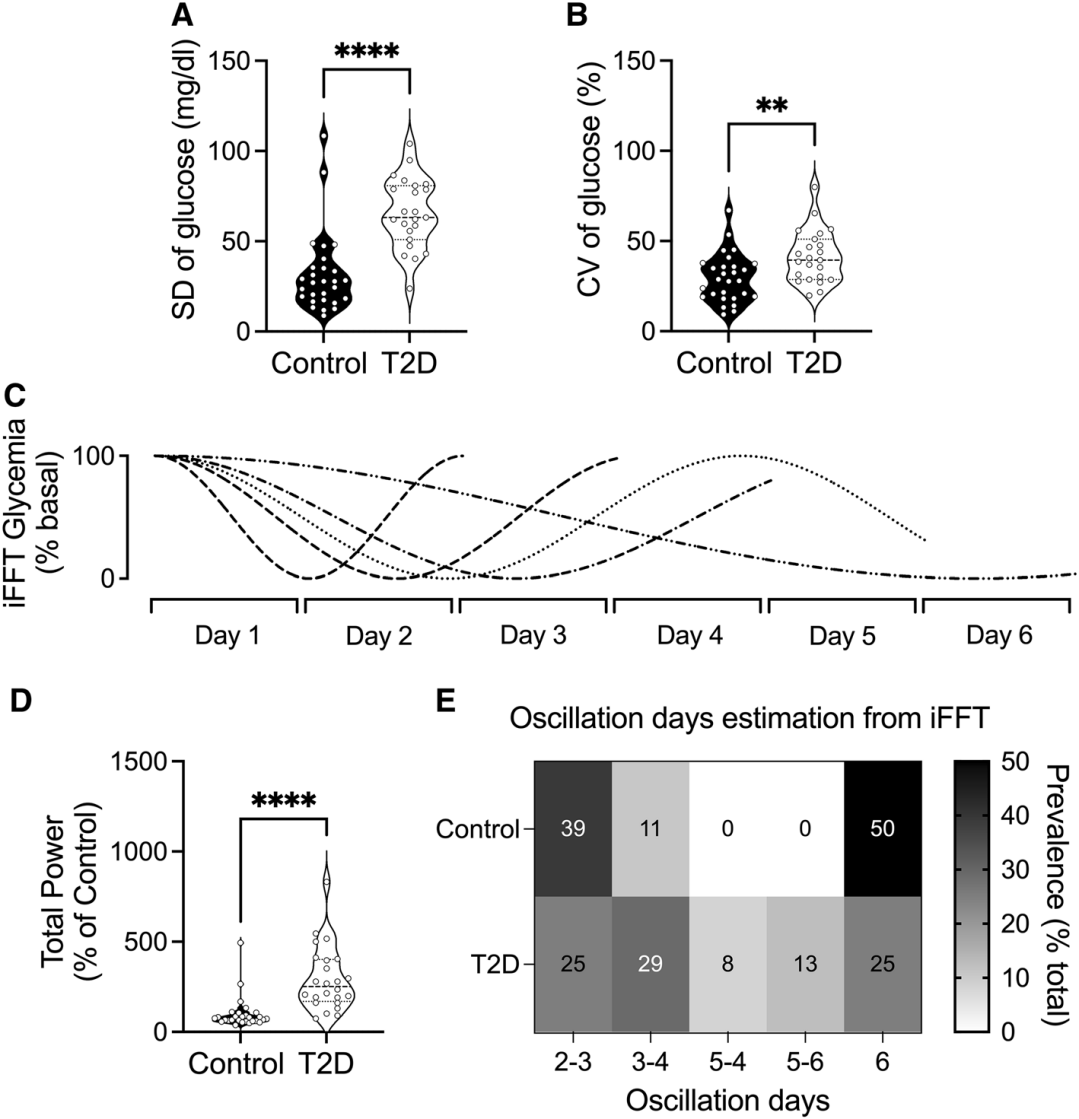
Glycemic variability in T2D patients. **A**, **B** Measurement of glycemic variability indices in patients with T2D and controls: SD, standard variation; CV, coefficientvariationtion. **C** Representative reconstruction from inverse Fast-Fourier Transform (IFFT) of maximum energy glycemic oscillation and real oscillation of glycemia at 2, 3, 4, 5, and 6 days, and **D** Total power. **E** Prevalence of maximum energy oscillation of glycemia in patients with T2D and controls. **, p < 0.01; ****, p < 0.0001. Control, n = 24; T2D, n = 28

Glucose variability assessed through the SD and CV indices of the CGM data is shown in Fig. 2. In contrast to the control group, the T2D group displayed a marked increase in both SD and CV (SD, p < 0.0001; and CV, p < 0.01, T2D vs. control, respectively) (Fig. 2A, B).

In addition to the traditional glycemic variability indices (SD and CV), we used FFT to determine the glycemic oscillatory pattern in patients with T2D. Results showed that the total power of glycemia was significantly higher in the T2D group than in the control group (p < 0.0001) (Fig. 2C, D). In addition, from the CGM data, we observed that the maximum phase glycemia oscillation varied from 2 to 6 days (Fig. 2C–E).

Our analysis revealed that the distribution of oscillation days (360º) was significantly different between groups (Chi-squared: 9.15; p = 0.032). The control group was mainly distributed at 2–3 and 6 days, whereas the T2D group showed a more homogeneous distribution across 2–3 -to 6 days (Fig. 2E).

### The predictive model of hyperglycemia and hypoglycemia in patients with T2D

**Fig. 3:**
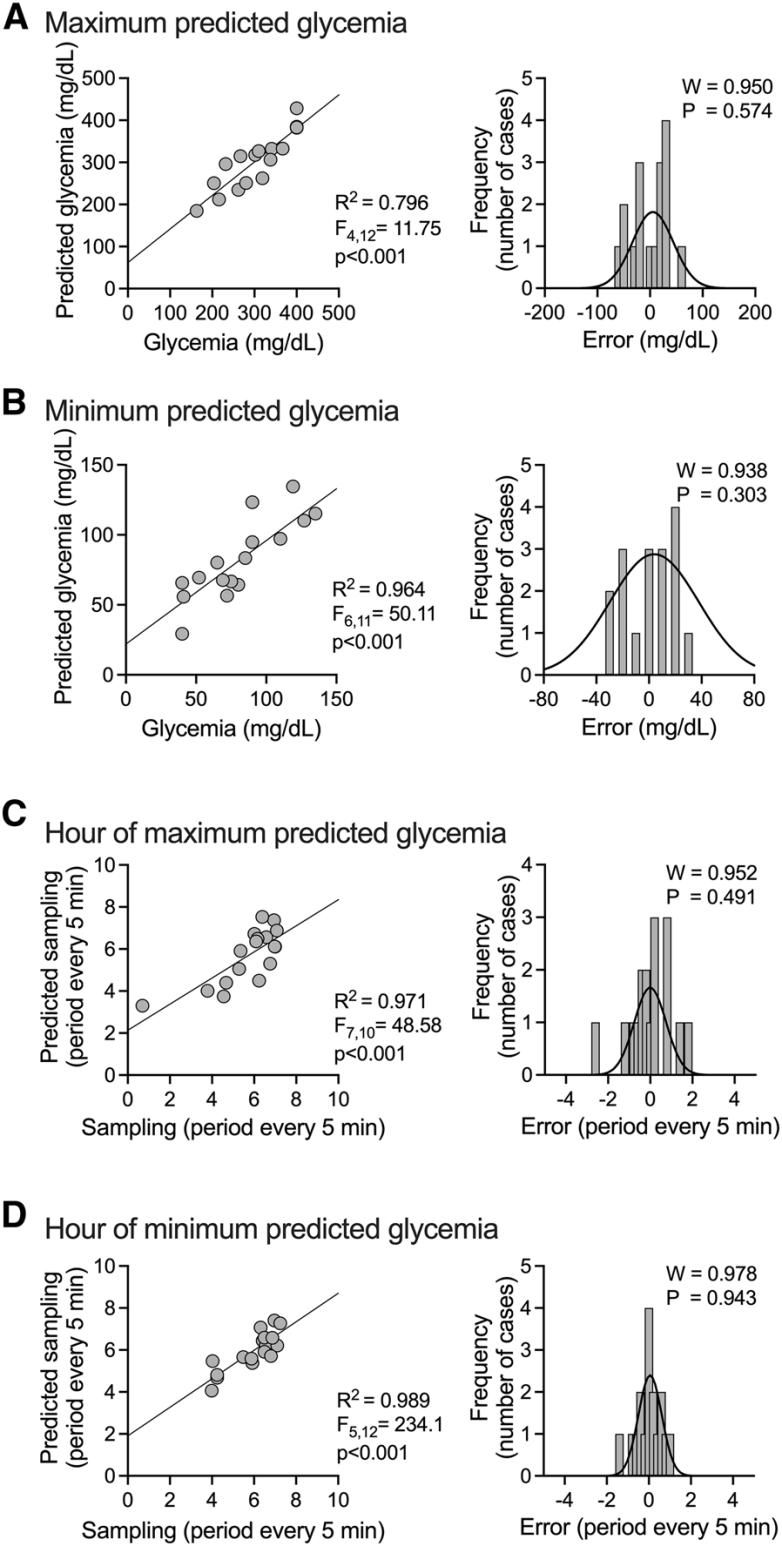
The predictive model of maximum and minimum glycemia oscillation in T2D patients. **A**, **B** (left panel) Scatterplot of real maximum and minimum glycemia (respectively) and predicted maximum and minimum glycemia (respectively); (right panel) error distribution of the model for maximum and minimum glycemia, respectively. The distribution of the error complies with the assumption of normality. **C**, **D** (left panel) Hour of maximum and minimum predicted glycemia (respectively); (right panel) error distribution of the model for an hour of maximum and minimum glycemia (respectively)

We recently developed a predictive model to determine maximum and minimum glycemic oscillations and the hour of the day at maximum and minimum glycemic oscillations in patients with T1D [15]; given that patients with T2D display different oscillatory days, we used the same model to test whether it could apply to T2D. Our data indicated that it was possible to predict hyperglycemia (p < 0.001) and hypoglycemia (p < 0.001) in patients with T2D (Fig. 3A, B left panel, respectively). The errors followed a normal distribution for both hyperglycemia (W = 0.95; p = 0.57) and hypoglycemia (W = 0.93; p = 0.30) (Fig. 3A, B right panel, respectively). In addition, the model was able to predict the hour in which the hyperglycemia (p < 0.001) and hypoglycemia (p < 0.001) occurred (Fig. 3C, D right panel, respectively). The time at which hyperglycemia and hypoglycemia occurred showed a normal distribution (W = 0.95; p = 0.49; and W = 0.97; p = 0.94, for hyperglycemia and hypoglycemia events, respectively) (Fig. 3C, D right panel, respectively).

## Discussion

The main aim of our study was to determine the characteristics of glycemic oscillation in patients with T2D patients and, if possible, predict dynamic glycemic variability over time. The principal results of our work were: (i) patients with TD2 have higher daily levels of HbA1c and glycemia; (ii) glycemic variability is greater in patients with TD2 than in controls; (iii) days of glucose oscillation are homogeneously distributed across six days in patients with T2D, and (iv) it is possible to predict the day time and magnitude of hyper- and hypoglycemic events in patients with T2D. Our finding that patients with T2D exhibit specific and predictable glycemic oscillatory patterns might have relevance for improving drug delivery and treatments related to glycemic control in T2D (i.e., insulin delivery, daytime physical exercise as treatment, among others).

It is well known that T2D pathophysiology is closely related to overweight and obesity [30, 31]. Estimations from the World Health Organization show that nearly 2 billion adults have overweight or obese [32], and between 50.9 and 98.6% of patients with T2D in Europe and 56.1% in Asia are obese [33]. Our data show that patients with T2D have overweight (assessed through BMI), which is accompanied by higher values of HbA1c, daily glycemia, and glycemic variability. Hyperglycemia in T2D can result from insulin resistance, lower insulin secretion, and/or reduced β-cell mass [34]. Of note, hyperglycemia is related to high glycemic variability [26]. While our results show that patients with T2D have higher sustained levels of glycemia and glycemic variability, we cannot discard the role of overweight per se on glycemic variability, as all patients displayed overweight and were diagnosed with T2D. The distinction between those variables was beyond the scope of our study, and future research needs to examine this critical point.

Contrary to our observations in T1D [15]. The present data reveal that glycemic oscillation was predominantly stationary (deterministic) in T2D but without significant differences between patients with T2D and controls. We previously showed that while patients with T1D displayed a predominance of stationary patterns, they were, nevertheless, different from those in control individuals [15]. These differences could be related to DM etiology, but our experimental design could not explain this discrepancy. Our data indicate that patients with T2D mainly exhibit deterministic behavior, suggesting that glycemic patterns could be predicted, similar to T1D [15, 35, 36]. Our data also reveal that patients with T2D show a marked increase in HbA1c, which has been associated with hyperglycemia but not glycemic variability. Indeed, although we have found a parallel increment of HbA1c and glycemic variability (SD, CV, and total power of glycemic oscillations) in T1D [15] and T2D (present data), it has been proposed that glycemic variability could be independent of HbA1c [37, 38]. In addition, HbA1c is thought not to be a robust measure of glycemic variability associated with hyper- and hypoglycemia events during the day and night phases [15, 37–39]. Therefore, while HbA1c is a classical clinical measure of long-term glycemic status and aids in diagnosing DM, it will likely be necessary to incorporate new analyses and measures considering circadian rhythms, to improve the personalized treatments against T2D.

Our results revealed that the distribution of oscillation differed between the groups. We observed that patients with T2D showed a homogeneous distribution across 2 to 6 days, which was different from the control group, which exhibited a heterogeneous distribution concentrated at 2–3 days and six days. Thus, our FFT-based mathematical apparently could discriminate between circadian and non-circadian oscillators. Nevertheless, considering the origin of our model, this is classified as a “closed loop,” which limits the extrapolation of our results. Similarly, the UVA/Padova system [40], although using machine learning and artificial intelligence, has reported similar limitations associated with long-term extrapolation. However, despite this limitation, our present data and our previous results [15], it is possible to propose that in the context of DM, the circadian rhythm of glycemic control becomes more evident, making it possible to predict regardless of etiology. Alterations in circadian rhythms might contribute to the pathogenesis of T2D [41, 42]. Gabriel et al. (2021) recently proposed that circadian rhythms are generated by a self-regulating feedback loop of activators and transcriptional repressors. Along this line, clarifying whether alterations of the circadian rhythm of some "clock genes," which could be associated with metabolic dysfunction in T2D and defining, for example, alterations in mitochondrial physiology, might be relevant to mechanistically determine the role of circadian rhythm on glycemic control in T2D [44]. Indeed, bidirectional communication between mitochondrial function and rhythmic gene expression has been identified and is altered in T2D [43]

## Strengths and limitations

The main strengths of the study are as follows: we determined that the glycemia signal in T2D exhibits a stationary pattern with homogeneous distribution across 2 to 6 oscillation days, and we tested a predictive model to assess hyper- and hypoglycemia and the hour at which these events occur in T2D patients. These findings may contribute to optimizing health spending associated with this pathophysiological condition, as there are critical hours where hyper- and hypoglycemia occur, which could be relevant to improving personalized therapies against T2D. One of the more important messages of this study is that it is possible to determine glycemic dynamic oscillations, which display characteristics of circadian patterns in T2D. However, deciding whether this circadian oscillatory pattern is related to medications, insulin delivery, and/or programmed physical exercise will be essential. In addition, our results do not dispose the possibility that glycemic oscillation could be secondary to poor glycemic control (i.e., elevated HbA1c). Therefore, future studies focus on patients with high glycemic variability will separate the role of poor glycemic control from intrinsic glycemic variability on the disruption of the circadian rhythm. Another limitation is the sample size, which could negatively impact our results. Finally, we tested a model used in T1D [15], which could influence our results. Therefore, future studies should develop a model more in line with the physiology of T2D.

## Conclusion

Based on CGM data, we found that patients with T2D exhibit a dynamic glycemic fluctuation with a specific oscillatory pattern showing circadian characteristics. Considering that the glycemic oscillatory pattern was mainly stationary, this characteristic could help to develop new therapeutic strategies and/or create a system alert to improve the outcome of this critical population.

## Materials and methods

### Ethics

We performed a single-center retrospective study with 28 adult controls and 24 patients with T2D. All participants signed an informed consent according to CIOMS Guideline #4 [23]. The study methods and experimental protocols were approved by the Ethical Committee of the Clinical Santa Maria, Santiago, Chile (approved #14) and were performed according to the principles of the Declaration of Helsinki.

### Study population

Fifty-two patients between 46 and 81 years of age were divided into two groups: 28 controls (53.84%) and 24 patients with T2D (46.15%). An endocrinology specialist recruited patients from Clinical Santa Maria according to the following exclusion criteria: (i) gestational diabetes; (ii) < 6 days of continuous blood glucose monitoring; (iii) < 18 years old; and (iv) female participants should not be in the first seven days of the follicular phase (v) no clinical records. The baseline characteristics of the participants are listed in Table 1.

### Intervention and procedures

CGM was performed using a glucose monitoring device (MiniMed iPro2), Medtronic Inc., Northridge, CA) with an Enlite sensor (both from Medtronic Inc., Northridge, CA), which consisted of a transmitter and receiver, and a disposable subcutaneous glucose sensor containing glucose oxidase, an enzyme that catalyzes the electrochemical reaction between glucose and oxygen, obtaining an electric current in nanoamps [24]. The sensor was inserted under the skin of the non-dominant arm and was programmed for interstitial glucose measurement every 5 min.

### Calibration and data collection

A calibration algorithm was used for the CGM system following the manufacturer's guidelines. From continuous recordings, HbA1c was determined according to the following formula: HbA1c % = (Avg glucose + 46.7)/28.7; after HbA1c mmol/mol = (10.93 * HbA1c %)—23.5, where Avg glucose is the arithmetic mean of the glucose calculated over all the measurements from the CGM. Calculations were performed with CareLink iPro software (version 2.2.005, Northridge, CA) [25]. Interstitial glucose measurements obtained after each hour were averaged, generating 24 data points per day across the six days of the study, and reported as mg/dL. Glucose variability parameters were calculated using the standard deviation (SD) and the coefficient of variation (CV) indices [26]. The SD (from 6 days) was divided by the arithmetic mean (from 6 days) of the corresponding glucose reading to determine the CV. Written instructions regarding food consumption times were provided to all participants. Food consumption times included breakfast (07:00–10:00 h), lunch (12:30–15:00 h), dinner (19:00–22:00 h) and post-dinner (22:30–00:00 h). The suggested caloric intake included (of total daily energy intake) 45–65% of carbohydrates, 15–20% of protein, and < 30% fat, in addition to 25–50 g/day of fiber [27]. Caloric intake was divided into breakfast, lunch, dinner, and post-dinner. To sure these instructions, we called every day to the patients aiming to remember all instructions/suggestions. In addition, we used a checklist to check every point. To determine the possible effects of pharmacological treatments in both groups, we assessed the prevalence of the main medications each patient received, considering that patients followed a scheme provided by the diabetes unit. Patients with T2D were administrated standard insulin before breakfast and ultrafast insulin before every food intake. The treatment was divided into three groups: rapid-acting insulin, long-acting insulin, and other hypoglycemic drugs, represented with "n" and % in each experimental group. The patients did not receive any other medications that could affect glycemic control. All participants were strongly advised not to engage in or perform any physical exercise other than their daily work-home activities; however, the daily activities were not restricted.

### Anthropometric variables and medical history

Selected patients were interviewed to assess their family and personal history of metabolic and cardiovascular diseases and their self-care activities associated with glycemic control (glucose management, dietary control, and physical activity). After the interview, height (m) and body mass (kg) were measured to determine body mass index (BMI) (kg/m^2^). The prevalence of euglycemia, hyperglycemia, and hypoglycemia were determined in all experimental groups through CGM.

### Glycemic oscillatory patterns

Based on previous studies, the circadian rhythm oscillation of all patients was determined by the Fast Fourier Transform (FFT) algorithm to obtain frequency oscillatory waves of glycemia [15]. For the present data structure, each blood glucose signal corresponds to 1,440 data points (including all data points), and the missing data in the signal was replaced by the minimum energy oscillation reconstruction or extrapolation accordingly to the maximum energy oscillation. The FFT algorithm was applied to obtain different frequencies, and the functions provided by the FFT (NUMPY package, Python Anaconda 3.6.6, 64-bit version; Python Software Foundation, Amsterdam, The Netherlands) were used to calculate the power spectral density (PSD). Subsequently, the inverse FFT IFFTT) was used to verify the quality of the glycemic signal. Then, the frequency with the highest power (over 15,000 a.u.) was selected based on the energy weight signal, and the other oscillation waves were discarded (< 24 h, ultra-circadian oscillation). The total power of the signal and the frequency at the maximum PSD were plotted (circadian oscillation). To determine the incidence of different circadian oscillations in all participants, the data were divided into > 2 to < 3; > 3 to < 4; > 4 to < 5; > 5 to < 6; and 6 days to reach maximum oscillation. The analysis was performed using Python Anaconda 3.6.6, a 64-bit version.

### Stationary/non-stationary CGM analysis

The Dickey-Fuller test was used to determine the stationary and non-stationary patterns of glucose variability from the time series provided by the CGM system, as described [15]. This test is a non-linear estimation and assumes that the data and the previous data point (delay time) are interdependent. The following formula was used to determine the stationary and non-stationary modes: yt = pyt-1 + ut. Where y is the glucose data, t is the time, and p is the constant coefficient associated with the autoregressive analysis. Stationary variables are defined as variables with no significant (p > 0.05) change in variance at any time. By contrast, non-stationary variables are defined as variables that change significantly over time (p < 0.05). We used R software (R-Project, Vienna, Austria) for analysis.

### The predictive model of glycemia

Our predictive model resulted from the convergence of several successive steps to find the best linear model that shows higher and more robust adjustments [15]. Therefore, our algorithm was applied as follows: i) Apply natural logarithmic transformation to the time of minimum and maximum glycemia; and ii) establish a linear regression between the minimum and maximum glycemia and minimum and maximum glycemic time and all FFT weights. The first 140 data points are used for adjustment and are randomly selected. This operation was repeated at least 40 times for each model to identify points that may cause problems in multiple adjustment models. The remaining data were used to calculate the fit of the final model. The best model was selected using the Akaike criterion [28]. The non-significant variables were eliminated individually, leaving only the significant ones (p < 0.05). The analysis was performed using Python software, version 3.8.1.

### Statistical analyses

Data are expressed as mean ± SD or 95% confidence interval (glycemic oscillatory pattern data). All data were subjected to normality (Shapiro–Wilk) and homoscedasticity (Levene) testing. Data were evaluated using an unpaired T-test (T2D vs. control) or analysis of variance (ANOVA), followed by the Holm-Sidak post- hoc test for CGM data analysis. Non-normal variables were evaluated using the Mann–Whitney test. To determine the distribution days, Chi-squared analysis was used. A p-value < 0.05 was considered statistically significant. All analyses were performed with GraphPad Prism 9.0 (La Jolla, CA, USA) and R software.

## Data Availability

The datasets generated and/or analyzed during the current study are not publicly available due that can be used in electronic devices but are available from the corresponding author upon reasonable request.
